# High-throughput screening to engineer optimal T cell therapies: current knowledge and future prospects

**DOI:** 10.3389/fonc.2026.1787786

**Published:** 2026-04-16

**Authors:** Lei Liu, Saisai Chen, Meng Ding, Yuqing Duan, Lingling Pu, Liang Li, Hui Peng, Qingsheng Yu

**Affiliations:** 1 Department of General Surgery, Fuyang Hospital of Traditional Chinese Medicine, Fuyang, China; 2 Department of General Surgery, Taihe Hospital of Traditional Chinese Medicine Affiliated to Anhui University of Chinese Medicine, Fuyang, China; 3 Department of General Surgery, The First Affiliated Hospital of Anhui University of Chinese Medicine, Hefei, China; 4 Graduate School, Anhui University of Chinese Medicine, Hefei, China

**Keywords:** adoptive T cell therapies, cells, CRISPR screening, genetic screening, high-throughput screening, non-genetic screening, TCR-T cells

## Abstract

Adoptive T cell therapy (ACT) is a potent strategy in cancer immunotherapy, but its clinical efficacy is often limited by primary resistance. To overcome this challenge, high-throughput screening technologies have emerged as essential tools for optimizing ACT. By enabling the identification of biologically significant targets and substances from vast libraries, these technologies have accelerated the development of advanced ACT strategies. This review delves into the latest advancements in high-throughput screening, highlighting its applications in genetic screening of T cells and tumor cells, as well as non-genetic screening for small molecules and targeted delivery systems. These insights provide valuable guidance for future research and clinical applications of ACT.

## 1 Introduction

The field of oncology has been transformed by the rapid evolution of immunotherapy, which hinges on the intricate crosstalk between the immune system and malignant cells (1). A cornerstone of this approach is Adoptive Cell Transfer (ACT)—a strategy wherein immune cells are harvested from patients or donors, expanded or genetically reprogrammed ex vivo, and subsequently reinfused to bolster anti-tumor immunity (2). Among these, T cell-centric modalities, including Tumor-Infiltrating Lymphocytes (TILs), TCR-engineered T cells, and CAR-T cells, have demonstrated remarkable clinical success in hematological malignancies (3). However, adoptive T cell therapy is not always effective against certain hematologic malignancies and most solid tumors, often leading to the development of primary resistance (4, 5). This therapeutic failure is driven by multifaceted mechanisms, such as impaired T cell expansion, diminished cytotoxic potency, the onset of exhaustion, and tumor-driven immune evasion through antigen loss or intrinsic inhibitory pathways (6). Consequently, deciphering the regulatory signals that govern T cell-tumor interactions is paramount to optimizing cellular therapies.

Genetic screening, which involves designing and integrating large gene-editing libraries into cell pools, allows for the identification of biologically important targets, later verified through functional assays (7). This method overcomes the limitations of single-gene editing, greatly advancing cancer genomics insights. The CRISPR/Cas9 (Clustered Regularly Interspaced Short Palindromic Repeats/CRISPR-associated 9) system has become the foremost high-throughput gene screening technique due to its flexibility and editing efficiency (8). With ongoing advancements, CRISPR-based screens using non-double-strand-breaking technologies like base editors and epigenetic editors are emerging to overcome certain technological challenges and are widely used in adoptive T cell therapy and related strategies. The synergy between genetic and non-genetic screening platforms provides a more holistic understanding of synergistic drug combinations and precision delivery systems (9). This review examines state-of-the-art high-throughput screening methodologies and evaluates their contemporary utility in characterizing T-cell dynamics, tumor responses under immune-mediated stress, and emerging combinatorial strategies, providing a roadmap for future clinical translation.

## 2 Advanced high-throughput screening technologies

### 2.1 Genetic screening

#### 2.1.1 Mechanism and characteristics

Genetic screening represents a robust framework for unearthing novel biological targets and regulatory circuits, owing to its exceptional throughput and operational efficiency. This methodology encompasses RNA interference (RNAi) via siRNA and shRNA, alongside the transformative CRISPR/Cas9 screening platforms. By deploying comprehensive libraries targeted at the whole genome or specific functional subsets, researchers can induce genetic perturbations in target populations, such as T cells and malignant cells. These experimental screens are conducted under diverse conditions, ranging from *in vitro* T-cell/tumor co-cultures and chronic pharmaceutical challenges to *in vivo* validation within immunocompetent or immunodeficient NSG murine models. These setups often include blank or conditional controls, and sometimes utilize survival pressure for comparative analysis. If gene editing leads to changes in cell phenotypes, Fluorescence-Activated Cell Sorting (FACS) can be employed to classify cells based on different surface markers or cytokine production. Subsequently, high-throughput sequencing technology quantifies single guide RNA (sgRNA) or other gene editing elements within each sample. This involves reading barcodes after Polymerase Chain Reaction (PCR) amplification and analyzing the depletion or enrichment of sgRNAs in gene-edited cells (7).

**Figure 1:**
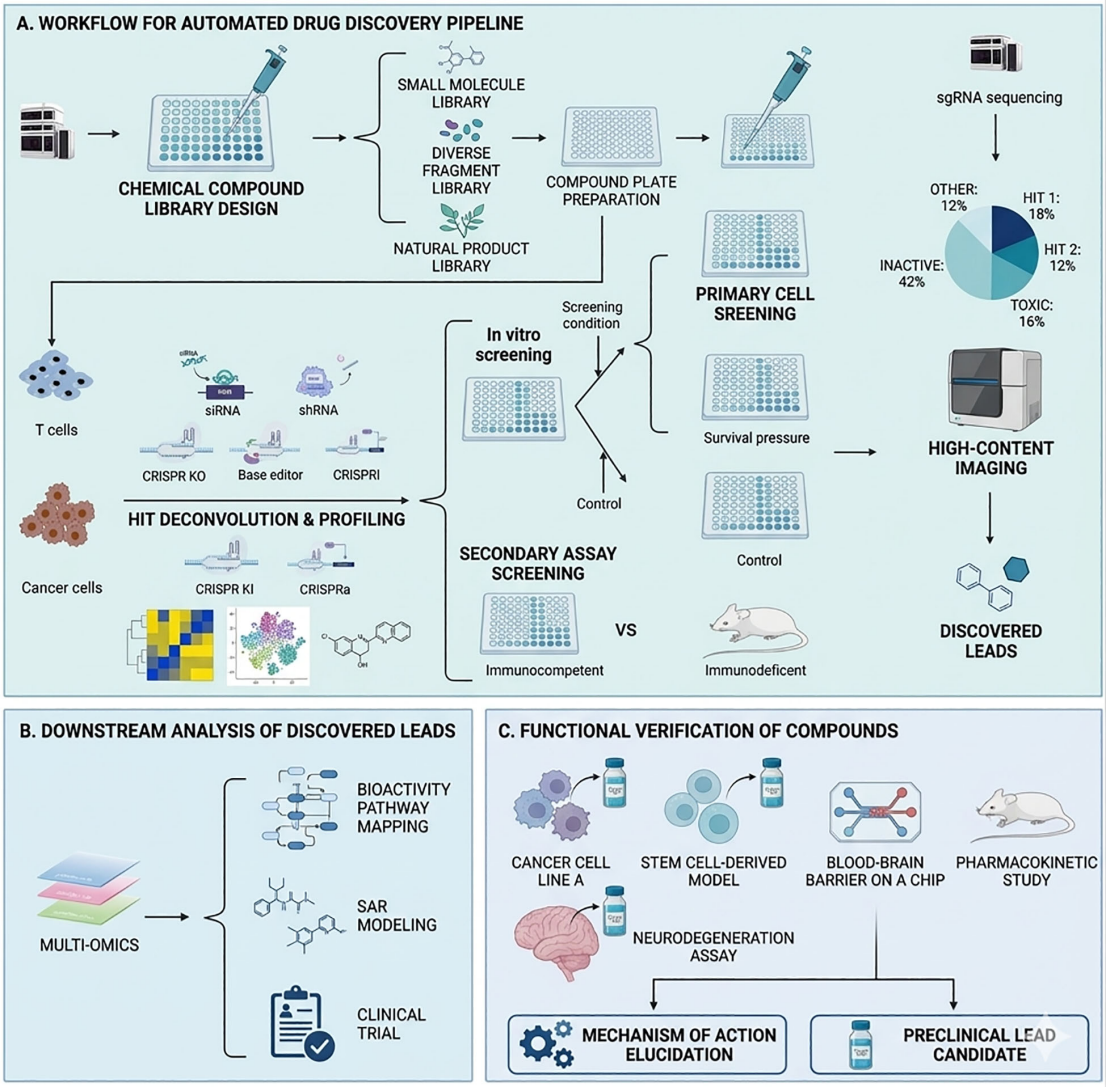
Advanced technologies in high-throughput genetic screening. **(A)** Libraries comprising siRNA, shRNA, and sgRNA were developed and introduced into tumor cells or T cells via viral vectors. The screening process was divided into loss-of-function (LOF) and gain-of-function (GOF) methodologies, employing siRNA, shRNA, CRISPR knockout (KO), base editing, or CRISPR interference (CRISPRi) for LOF, and CRISPR knock-in (KI) and CRISPR activation (CRISPRa) for GOF. *In vitro* screening involved assessing experimental groups against control groups under specific conditions, or sorting cells based on surface markers and other phenotypic traits using Fluorescence-Activated Cell Sorting (FACS). *In vivo* screening primarily compared immunocompetent and immunodeficient models, with target identification achieved through sgRNA sequencing. **(B)** The downstream analysis of the identified targets focused on gene set enrichment analysis (GSEA), multi-omics integration, and predictions for clinical outcomes. **(C)** Genetic modifications to screened targets in T cells and tumor cells, along with the use of activating or inhibitory compounds, improve the effectiveness of adoptive T cell therapy and other innovative combination strategies, thereby enhancing T cell functionality and tumor-killing capabilities.

Genetic screening strategies generally fall into two categories: loss-of-function (LOF) and gain-of-function (GOF) approaches. Conventional platforms, including siRNA, shRNA, and CRISPR/Cas9, primarily facilitate gene silencing or knockout to uncover resistance-conferring genes in tumor cells or negative regulators within the immune compartment. While LOF screens have significantly advanced cellular therapies, the capacity to integrate large gene cassettes for functional modification represents a more transformative frontier. Currently, GOF screening predominantly utilizes CRISPR activation (CRISPRa) to transiently upregulate transcription; however, the clinical utility of CRISPRa is often hampered by the bulky size and potential immunogenicity of Cas9-activator fusions. This underscores the necessity for robust CRISPR knock-in (KI) systems. Notably, Roth et al. pioneered a high-throughput KI library using barcoded, non-viral DNA templates to target specific loci in parallel. This breakthrough streamlined the identification of genes that bolster T-cell fitness and enabled precise, pooled GOF evaluations (10). Furthermore, the deployment of barcoded lentiviral open reading frame (ORF) libraries has made genome-scale GOF screening feasible in primary human T cells. When coupled with direct ORF capture and single-cell sequencing, these advancements provide high-dimensional insights that are essential for refining next-generation cancer immunotherapies (11) (**Figure 1A**).

Downstream analysis is a crucial phase following target identification, often employing gene set enrichment analysis (GSEA) to uncover functional pathways involved in T cell-mediated tumor cell cytotoxicity and to validate essential phenotypes related to these functions. For example, utilizing CRISPR/Cas9 for cellular modifications or small molecule drugs to adjust targets can help confirm their influence on positive outcomes such as T cell activation, differentiation, and tumor cytotoxicity, as well as on negative conditions like exhaustion (12, 13). This approach has made significant strides in T cell research, contributing to a deeper understanding of cellular responses and interactions (14–19) (**Figures 1B, C**).

#### 2.1.2 Emerging CRISPR screening tools

siRNA, which consists of artificially synthesized short RNA fragments, is typically limited to independent phenotype screening formats such as 96-well or 384-well culture plates. An alternative strategy involves utilizing commercial siRNA libraries, where each siRNA is assigned a unique barcode. The process of identifying effective siRNAs relies on quantifying these barcodes, which can be both limited in scope and operationally cumbersome (20). shRNA facilitates library screening through viral vectors, allowing for a more streamlined and efficient process with the aid of deep sequencing. However, the high-throughput use of RNA interference (RNAi) does not consistently achieve complete gene expression suppression needed for stable phenotypes, and it often presents higher off-target effects, which can undermine its accuracy in practical applications (21–23).

Traditional CRISPR/Cas9 genome editing induces targeted DNA double-strand breaks (DSBs), which can lead to numerous indels and genetic instability. In contrast, deactivated Cas9 (dCas9) and nickase Cas9 (nCas9) effectively prevent DSBs, thereby reducing the risks associated with genetic mutations. The development of DNA Base Editors (BEs) has shifted the focus from gene deletion to precise nucleotide replacement. Utilizing sgRNA-guided deaminases, BEs enable targeted transitions that help researchers dissect the functional impact of specific codons in immune-related genes (24–27). To complement this, CRISPRi and CRISPRa systems provide a non-integrative means of regulating gene expression. By localizing transcriptional modulators to the Transcription start site (TSS), these systems can either silence or bolster endogenous transcripts without permanently altering the DNA sequence (28). This reversibility reduces the risk of unintended cellular stress and allows for the study of genes that may be inactive in resting states. By utilizing both inhibitory and activating screens, researchers can more accurately map the complex regulatory networks that govern T-cell and tumor-cell interactions (15, 29–31).

#### 2.1.3 RNA interference–based gene silencing

Both siRNA and shRNA operate through the endogenous RNA interference (RNAi) pathway, a conserved post-transcriptional regulatory system that controls gene expression through sequence-specific degradation of messenger RNA (mRNA). In mammalian cells, RNAi-mediated silencing is executed by the RNA-induced silencing complex (RISC), whose catalytic core contains members of the Argonaute protein family, particularly AGO2. Synthetic siRNAs are typically introduced directly into the cytoplasm as short double-stranded RNA molecules (~21–23 nucleotides). Once inside the cell, the duplex is incorporated into RISC, where the passenger strand is removed while the guide strand remains associated with AGO2. Guided by base-pair complementarity, the AGO2–siRNA complex binds to the target mRNA and induces endonucleolytic cleavage, leading to rapid degradation of the transcript and suppression of protein translation. Because synthetic siRNA molecules are not genomically integrated and are progressively degraded or diluted during cell division, the resulting gene silencing is transient, typically lasting several days. This short-term perturbation makes siRNA particularly useful for arrayed functional screens performed in multi-well plate formats.

In contrast, shRNA-based RNAi provides a more stable mode of gene knockdown. shRNA constructs are commonly delivered via integrating viral vectors such as lentiviruses, enabling sustained intracellular expression of short hairpin transcripts driven by RNA polymerase III promoters (for example U6 or H1). Following transcription in the nucleus, shRNA molecules undergo sequential processing by components of the endogenous microRNA machinery. The hairpin precursor is initially recognized by the Drosha–DGCR8 microprocessor complex, exported to the cytoplasm through Exportin-5, and subsequently cleaved by the RNase III enzyme Dicer to generate a siRNA-like duplex. The processed guide strand is then loaded into RISC, where it mediates sequence-specific mRNA degradation in a manner analogous to siRNA. Because the shRNA expression cassette is stably integrated into the host genome, the resulting knockdown can persist for extended periods—often weeks or across multiple cell divisions—which makes shRNA particularly suitable for pooled loss-of-function screening under long-term selection pressures. Nevertheless, RNAi-based approaches typically produce partial gene suppression rather than complete ablation, and off-target effects may arise from imperfect guide–target complementarity.

#### 2.1.4 CRISPR–Cas9 genome editing

In contrast to RNAi-based technologies, CRISPR/Cas9 systems perturb gene function at the DNA level, thereby enabling more durable and often permanent genetic modifications. The canonical CRISPR/Cas9 editing platform consists of two essential components: a single-guide RNA (sgRNA) that directs sequence-specific DNA recognition, and the Cas9 endonuclease, which introduces a site-specific double-strand break (DSB) at the targeted genomic locus adjacent to a protospacer-adjacent motif (PAM).

Following cleavage, the DSB is resolved by the cell’s endogenous DNA repair machinery, primarily through one of two pathways. The first, non-homologous end joining (NHEJ), is an error-prone repair process that frequently introduces small insertions or deletions (indels) at the break site, often disrupting the coding sequence and producing functional gene knockouts. Alternatively, in the presence of an exogenous donor template, cells can repair the break via homology-directed repair (HDR), allowing the precise insertion or replacement of defined DNA sequences. Because these alterations are incorporated directly into the genome, CRISPR-mediated modifications are generally permanent and heritable during cell division, providing a robust platform for genome-scale pooled screening.

Recent innovations have further expanded the versatility of CRISPR-based perturbation systems. Catalytically inactive dead Cas9 (dCas9) can be fused to transcriptional regulators to modulate gene expression without introducing DNA breaks. In this configuration, CRISPR interference (CRISPRi) recruits transcriptional repressors to promoter regions to inhibit transcription, whereas CRISPR activation (CRISPRa) recruits transcriptional activators to enhance endogenous gene expression. In addition, next-generation tools such as base editors and prime editors enable precise nucleotide substitutions without double-strand break formation, providing new opportunities to dissect the functional consequences of specific coding variants in immune regulatory genes.

#### 2.1.5 Implications for functional genetic screening

The mechanistic differences between RNAi- and CRISPR-based perturbation platforms directly influence their utility in functional genetic screening. RNAi technologies offer rapid and reversible modulation of gene expression, making them useful for probing genes whose complete deletion may be lethal. By contrast, CRISPR-based systems enable stable gene disruption or targeted genome engineering, which facilitates long-term selection experiments and the identification of genes that control cellular fitness, immune signaling, or tumor resistance pathways.

In the context of cancer immunology and T-cell engineering, these complementary technologies have enabled systematic mapping of regulatory networks that govern T-cell activation, differentiation, exhaustion, and tumor cytotoxicity. Coupled with high-throughput sequencing readouts, pooled screening libraries allow researchers to quantify the enrichment or depletion of specific genetic perturbations across experimental conditions, thereby revealing candidate regulators that shape immune–tumor interactions.

### 2.2 Non-genetic screening

In adoptive T cell therapy, the combination of genetic and non-genetic screening underpins both combination therapies and affinity studies. Comprehensive drug sensitivity screening reveals the genomic mechanisms behind the synergy of drug treatments with tumor immunotherapy. In this process, tumor cells tagged with luciferase (Luc) are co-cultured with T cells and a library of drugs in formats such as 96-well or 384-well plates. By assessing Luc activity, researchers can identify effective drugs that enhance the efficacy of adoptive T cell therapy through improved tumor cell killing. Subsequently, CRISPR screening is employed to determine specific mechanisms, helping to reveal the immunomodulatory properties of various cancer therapies.

Non-genetic screening for non-viral delivery vectors often utilizes expansive chemical libraries to optimize delivery performance. Initial evaluations focus on the structural properties of lipid nanoparticles (LNPs), where techniques like chromatography and TEM are used to measure critical parameters, including pKa and surface charge. Subsequent phases transition into *in vivo* models to verify targeting accuracy and protein translation efficiency within specific tissues. This systematic optimization process allows for the identification of superior delivery structures, providing a robust experimental foundation for improving the tissue-specificity and overall potency of modern delivery systems.

## 3 Genetic screening applied for T cells

While high-throughput screening (HTS) has traditionally been viewed as a stochastic tool for target discovery, a meta-analysis of recent data reveals a convergence toward a unified engineering paradigm. Rather than treating identified genes as isolated “hits,” we propose that the success of Adoptive T-cell Therapy (ACT) is governed by three hierarchical regulatory axes: Intrinsic Fitness, the Tumor-Immune Interactome, and Metabolic Adaptation. This framework allows for the transition from descriptive cataloging to the rational design of “synthetic immunity,” where screening data informs the systemic reprogramming of T cell behavior across multiple biological layers.

### 3.1 Primary T cell screening with cytotoxic functions

Collectively, the screening strategies described above provide complementary tools for systematically interrogating the molecular determinants of T-cell function. Building on these technological platforms, recent studies have applied genome-scale perturbation screens directly in primary T cells to identify genes that regulate key antitumor phenotypes, including cytotoxic activity, proliferative capacity, and tumor infiltration.

The identification of regulators such as BATF, PRDM1, and c-Jun through genome-wide CRISPR screens represents more than a list of potency enhancers; it defines a core epigenetic circuit of persistence. Recent HTS data suggest that these factors do not act in isolation but converge to remodel the chromatin landscape, effectively “locking” T cells into a self-renewing, stem-like memory state. For instance, the discovery that PRDM1 deletion or BATF overexpression can arrest terminal differentiation indicates that the next generation of ACT will move toward epigenetic rheostat engineering, where screening hits are utilized to tune the longevity of the T cell pool against chronic antigen stimulation.

**Figure 2:**
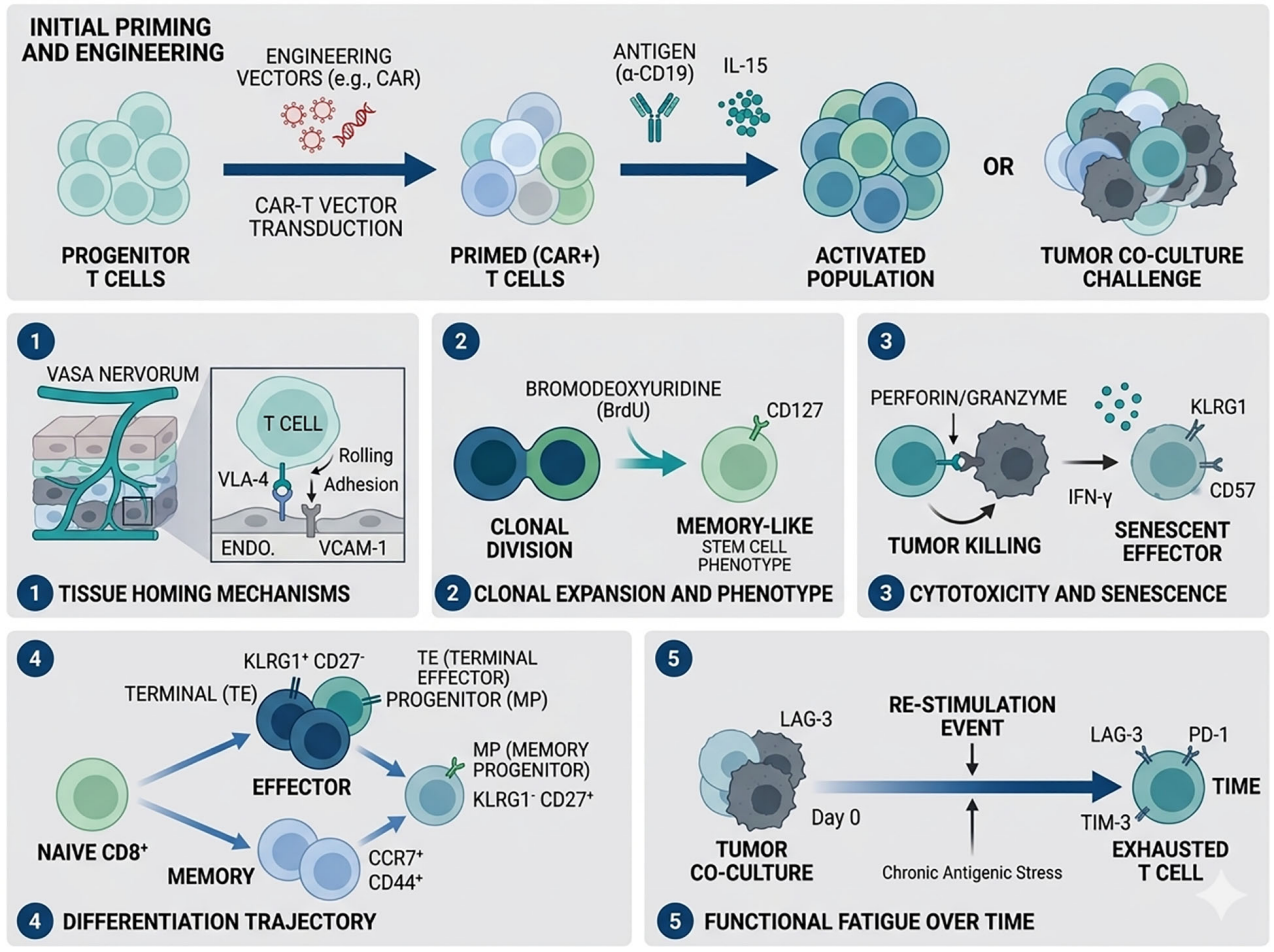
Screening of primary T cells for cytotoxic functions. Following the introduction of a viral library, primary T cells are modified through gene editing. Functional assessment of modified T cells involves TCR-mediated activation or tumor-cell challenge within optimized environments. Homing efficiency is evaluated through ICAM-1 screening or *in vivo* tracking, while proliferative fitness and early activation status are monitored via CFSE assays and CD69 levels. To define the effector profile, CD107a and IFN are utilized as definitive phenotypic indicators of lytic activity. Additionally, the Fas ligand system is essential for modulating programmed cell death. By analyzing the expression of CCR7 and CD62L, researchers can distinguish between EFF and MEM populations and pinpoint the factors that sustain the memory T-cell compartment. TEFF can be further categorized into short-lived effectors (TE; KLRG1+ CD127-) and memory precursor (MP; KLRG1- CD127+) cells. The latter group has the potential to differentiate into TMEM cells, providing long-lasting protective immunity against tumors. Ongoing *in vitro* experiments and longitudinal comparisons help pinpoint persistent targets in T cell therapy, particularly noting that exhausted T cells express inhibitory markers such as PD-1.

Phenotypic sorting is achieved using FACS, or cell populations subjected to various screening conditions are sequenced directly to identify genes that regulate specific functional phenotypes. Techniques such as single gene editing have been utilized to improve tumor-infiltrating lymphocyte (TIL) and CAR-T therapies, demonstrating enhanced success rates in treating tumors through adoptive T cell therapy, validated in both *in vitro* and *in vivo* settings (**Table 1**, **Figure 2**).

**Table 1:** Representative studies of genetic screening for T cells.

Screening method	Screening cells	Screening model	Library design	Screening strategies	Readout	Screened target	Function of screened target	Ref.
CRISPR KO	OT-IT cells	*in vivo*	Activates differentially expressed gene library between CD8 T cells and naïve CD8 T cells	T cells were injected into B16-OVA mice, and the distribution of T cells was observed	The comparison was in the lung/liver group, the stay in the circulation group, and the untreated group	St3gal1	St3gal1- βII-spectrin axis is the key cell-intrinsic program for cancer targeting CAR-T cell migration	(32)
CRISPR KO	OT-IT cells	*in vitro*	Gene library of PI3K-related pathways	TCR and scICAM were stimulated, and FACS sorted ICAM expression	The ICAM^pos^ group and the ICAM^neg^ group were compared	RASA3	RASA3 suppressed LFA-1 activation in T cells. .	(33)
CRISPR KO	OT-IT cells	*in vitro*	Genome-wide library	TCR stimulation, FACS sorting CFSE expression	Compare dividing T cells and non-dividing T cells	RASA2	RASA2 ablation enhanced MAPK signaling and CAR T cell cytolytic activity in response to target antigen	(34)
CRISPRi	Jurkat and primary human T cells	*in vitro*	Genome-wide library	TCR stimulation, FACS sorts GFP expression	Compare GFP^high^ and GFP^low^	NAMPT	NAMPT is required for T cell activation	(35)
CRISPRa	Primary human CD4^+^ and CD8^+^ T cells	*in vitro*	Human ORF Library	Pre-stimulation, FACS sorts CFSE expression	Compare Dividing and Undividing	LTBR	Induce transcriptional and epigenomic remodeling.	(11)
CRISPR KO	OT-I T cells	*in vitro*	Kinase-related gene library	TCR stimulation, FACS sorting cell proliferation, CD62L, ROS, γH2AX expression	The four phenotypic high-expression and low-expression groups were compared	P38	p38 kinase is a central regulator of all four phenotypes, including cell expansion, differentiation, oxidative stress, and genomic stress	(36)
CRISPRi	Jurkat t cells	*in vitro*	Genome-wide libraries	TCR stimulation, FACS sorting CD69	Compare CD69^high^ group and CD69^low^group	FAM49B	Inhibit T cell activation by repressing Rac activity and modulating cytoskeleton reorganization.	(37)
CRISPR KO	Primary human CD8^+^ T cells	*in vitro*	T cell activity-related gene libraries	TCR stimulation, FACS sorting TNF, IFN-γ, PD-1, CD25 expression	The production of TNF and IFN-γ was compared, and the high and low surface expressions of CD25 and PD-1 were compared	PIK3CD	Reduce T cell cytotoxic function.	(25)
CRISPR KO	OT-I T cells	*in vivo*	Genome-wide libraries	T cells are injected into Rag1- mice bearing E0771-mCh-OVA to observe T cell killing. T cells were co-cultured with SIINFEKL-pulsed E0771 cells, and FACS sorted CD107a expression	Direct readout to viable T cells *in vivo* The CD107a^high^ group and the CD107a^low^ group were compared	DHX37	Modulate NF-kB to suppress effector functions, cytokine production, and T cell activation	(38)
CRISPRa	OT-I T cells	*in vitro*	Genome-wide libraries	T cells were co-cultured with SIINFEKL-pulsed E0771 cells, and FACS sorted CD107a expression	The CD107ahigh group and the CD107alow group were compared	PRODH2	Enhance the metabolic and immune functions of CAR-T cells against cancer.	(30)
CRISPR KO	Primary human CD4^+^ T cells	*in vitro*	GPCR’s gene library	TCR activation and sorting of CD69, IL-2 and IFN-γ expression by FACS	The CD69 expression and high and low groups, and the number of IL-2 and IFN-γ produced were compared	S1PR1,GPR183	Similar to CXCR4 and contribute to T cell activation.	(39)
CRISPR KO	Jurkat T cells	*in vitro*	Genome-wide libraries	FASL stimulation	The control group and the FASL stimulation group were compared	EBF4	Induce cytotoxic T cell lysis	(40)
shRNA	CD8^+^ T cells	*in vitro*	shRNA libraries associated with chromatin	FACS sorts CD4 expression	CD4^+^ and CD4^-^ groups were compared	CAF-1	Stably repress expression of Cd4 gene.	(41)
CRISPRa and CRISPRi	Primary human CD8^+^ T cell	*in vitro*	A gene library of epigenetic modifiers associated with T cell status	FACS sorts CCR7 expression	The CCR7^high^ group and the CCR7^low^ group were compared	BATF3	Promote specific features of memory T cells.	(42)
CRISPR KO	OT-I T cells	*In vivo*	Metabolism-related gene libraries	Injected into mice, FACS sorts KLRG1^-^CD127^+^ (MP) and KLRG1^+^CD127^-^ (TE) phenotypes	The MP group, TE group and non-injected cell group were compared	Slc7a1, Slc38a2	Dampene the magnitude of TMEM differentiation through modulating mTORC1 signaling.	(43)
CRISPR KO	OT-I T cells	*in vivo*	A library that targets proteins involved in epigenetic modifications	Injected into mice, FACS sorts TE, MP, and normal phenotypes.	MP group, TE group and non-injected cell group were compared	cBAF	Inhibit T_mem_ cells formation	(44)
CRISPR KO	OT-I T cells	*in vivo*	Genome-wide libraries	TCR was repeatedly stimulated to observe T cell changes	IL-2 stimulation alone and α-CD3 and IL-2 stimulation were compared	INO80 Arid1a	Perturbation of the INO80 and BAF chromatin remodeling complexes improved T cell persistence.	(45)
CRISPR KO	OT-I T cells	*in vivo*	Transcription factor gene library	B16-OVA mice were injected to observe T cell survival	Direct sequencing reads after 7 days	IKAROS ETS1	The amplitude of T_MEM_ differentiation is modulated by modulating mTORC1 signaling.	(14)
CRISPR KO	CD8^+^ P14 cells	*in vitro*	TF library	Co-cultured with CD11C^+^DC cells for acute and chronic GP33 and IL-2 stimulation	T cells were compared on the second day versus the seventh day	BHLHE40	Regulate a key differentiation checkpoint between progenitor and intermediate Tex subsets	(46)
CRISPR KO	Primary human CD8^+^ T cell	*in vitro*	Epigenetic Regulator Library	Acute and chronic TCR stimulation was performed *in vitro*, and FACS sorted PD1^+^/TIM3^+^ expression	Acute and chronic stimulation groups were compared	mSWI/SNF	Perturbation of mSWI/SNF complexes enhances T cell persistence with attenuated exhaustion hallmarks and increased memory features.	(47)
CRISPR KO	68–41 T cells	*in vitro*	Genome-wide libraries	After *in vitro* culture, FACS sorts PD-1 expression	Compare PD-1^high^ and PD-1^low^	Fut8	Induce cell-surface expression of PD-1 and reduce T cell activation.	(48)

St3gal1,ST3 β-galactoside α-2,3-sialyltransferase 1;RASA3,Ras gtpase activating proteins3;RASA2,Ras gtpase activating proteins2; NAMPT, nicotinamide phosphoribosyltransferase; LTBR, lymphotoxin-β receptor;P38, a class of mitogen-activated protein kinases; FAM49B,family with sequence similarity 49 member B; PIK3CD, phosphoinositide-3-kinase catalytic subunit delta; DHX37, DEAH-Box Helicase 37; PRODH2,Proline Dehydrogenase 2;S1PR1, sphingosine-1-phosphate receptor;GPR183, G Protein-Coupled Receptor 183;EBF4, early B cell factor 4; CAF-1,chromatin assembly factor; BATF3, Basic leucine zipper transcription factor ATF-like 3; Slc7a1 and Slc38a2, amino acid transporters; cBAF, mammalian canonical Brg1/Brg-associated factor; INO80, INO80 Complex ATPase Subunit; Arid1a, AT-Rich Interaction Domain 1A; IKAROS, Family Zinc Finger Protein 2; ETS1, E-twenty six1; BHLHE40, Basic Helix-Loop-Helix Family Member E40; mSWI/SNF, mammalian SWItch/Sucrose Non-Fermentable; Fut8, Fucosyltransferase 8.

Adoptive T-cell therapies often struggle with poor tumor infiltration and organ entrapment following intravenous infusion, leading to reduced efficacy and safety concerns (49). Innovative *in vivo* screens have identified genes such as St3gal1 and βII-spectrin that are essential for directing T cells toward tumor tissues (32). The LFA-1/ICAM-1 axis is equally critical for effective cell adhesion (33). Screening efforts have demonstrated that knocking out RASA3 can optimize this migratory process (11). Proliferative capacity, measured by CFSE dye dilution, has also been used to find genes that improve CAR-T cell endurance; notably, RASA2 knockout enhances the effector profile of T cells in leukemia models (34). Finally, by leveraging CD69 as an activation marker, studies have identified FAM49B as a negative regulator that impairs T-cell responses through the disruption of Rac-dependent actin reorganization (37).

Essential to the specific cytotoxic functions of T cells, functional activation sites can be identified by targeting cytokines such as interferon-γ (IFN-γ) or by analyzing CD8+CD107a+ T cells (25, 30, 38, 39). For instance, Ye et al. identified gain-of-function (GOF) targets like Proline Dehydrogenase 2 (PRODH2) in CD8+CD107a+ T cells through CRISPRa screening. Incorporating PRODH2 into primary or CAR-T cells can modify metabolic pathways, leading to improved outcomes across various tumor types (30). Advanced base editing screening has also revealed alleles, including those for the phosphoinositide-3-kinase catalytic subunit delta (PIK3CD) protein, which can positively or negatively influence T cell cytotoxicity (25). In the context of T cell apoptosis, Fas-FasL (Factor-related Apoptosis ligand) signaling plays a critical role. Continuous stimulation of FasL, in contrast to normal controls, indicates that Early B cell factor 4 (EBF4) regulates the degradation of the anti-apoptotic protein c-FLIP, which can lead to the loss of cytotoxic T cells (40). The orchestration of T-cell subsets is a cornerstone of effective anti-tumor responses (41). Mechanistic studies using shRNA have shown that CD8+ T-cell stability requires the active repression of CD4 through chromatin remodeling (42). To optimize cellular therapies, researchers frequently monitor the expression of the lymphoid homing receptors CCR7 and CD62L, whose high expression is characteristic of naïve and central memory T cells and is commonly used to identify T-cell subsets with enhanced proliferative potential and long-term persistence (50). Functional genomic screens have highlighted BATF3 as a key transcription factor that increases the memory T cells (MEM), thereby enhancing the anti-tumor activity of engineered CAR-T cells (43, 44). Short-term effector cells (TE; KLRG1^+^ CD127^−^) and memory precursor cells (MP; KLRG1^−^ CD127^+^) are distinguishable, with TEs perishing within days to weeks, while MPs evolve into memory cells and provide prolonged protective tumor immune responses (51). Identifying the genetic drivers that push T cells toward this MP/memory fate is a major objective in modern immunotherapy research (48).

### 3.2 TCR-T and CAR-T cell directly screening

Direct genomic screening within TCR-T and CAR-T cell populations offers a powerful method for optimizing adoptive cell therapies. By introducing sgRNA libraries into engineered T cells and employing tumor co-culture assays, researchers can isolate genetic drivers of exhaustion and cytotoxicity through PD-1 or cytokine-based sorting. Key discoveries include SNX9, which regulates T-cell activation through PLC and NFATC2 (52), and TLE4, whose loss prevents CAR-T cell apoptosis (53). Additionally, IKZF2 has been identified as a regulator of NFAT-driven anti-tumor responses. Research into the Mediator complex subunits MED12 and CCNC further demonstrates how epigenetic shifts can be leveraged to strengthen CAR-T cell effectiveness (54). These high-throughput approaches streamline the identification of targets that enhance tumor-specific killing and provide robust models for evaluating long-term antigen challenge, thereby advancing the precision of immunotherapy.

Additionally, random integration into the host genome may disrupt target genes, leading to reduced screening accuracy and complicating data interpretation (9). To overcome these obstacles, researchers have developed innovative solutions. For instance, Dai and colleagues devised a system called CLASH, based on AAV-KIKO, which utilizes CRISPR/Cas12a technology. Using the CLASH platform, researchers analyzed CD22 CAR-T cells after extended co-culture both *in vitro* and *in vivo*, discovering that the exon 3 skip mutant of PR/SET Domain 1 (PRDM1) significantly enhances various anti-tumor effects (55). Additionally, Blaeschke and his team introduced a Modular Pooled Knock-In (ModPoKI) screening method, which combines functional modular libraries with sequences for NY-ESO-1 TCR, CD19-BBz CAR, or HA-GD2-28z attached to sgRNA. This technique allows for the generation of TCR-T and CAR-T cells at the TRAC locus and supports extensive CRISPR knock-in screening within cell pools. Following chronic, repetitive co-culturing and stimulation, they found that the overexpression of BATF and transcription factor AP4 (TFAP4) collaboratively influences TCR/CAR gene expression and the functionality of T cells, a finding that supports the context of anti-tumor immunity (56).

### 3.3 CARs structure screening

**Figure 3:**
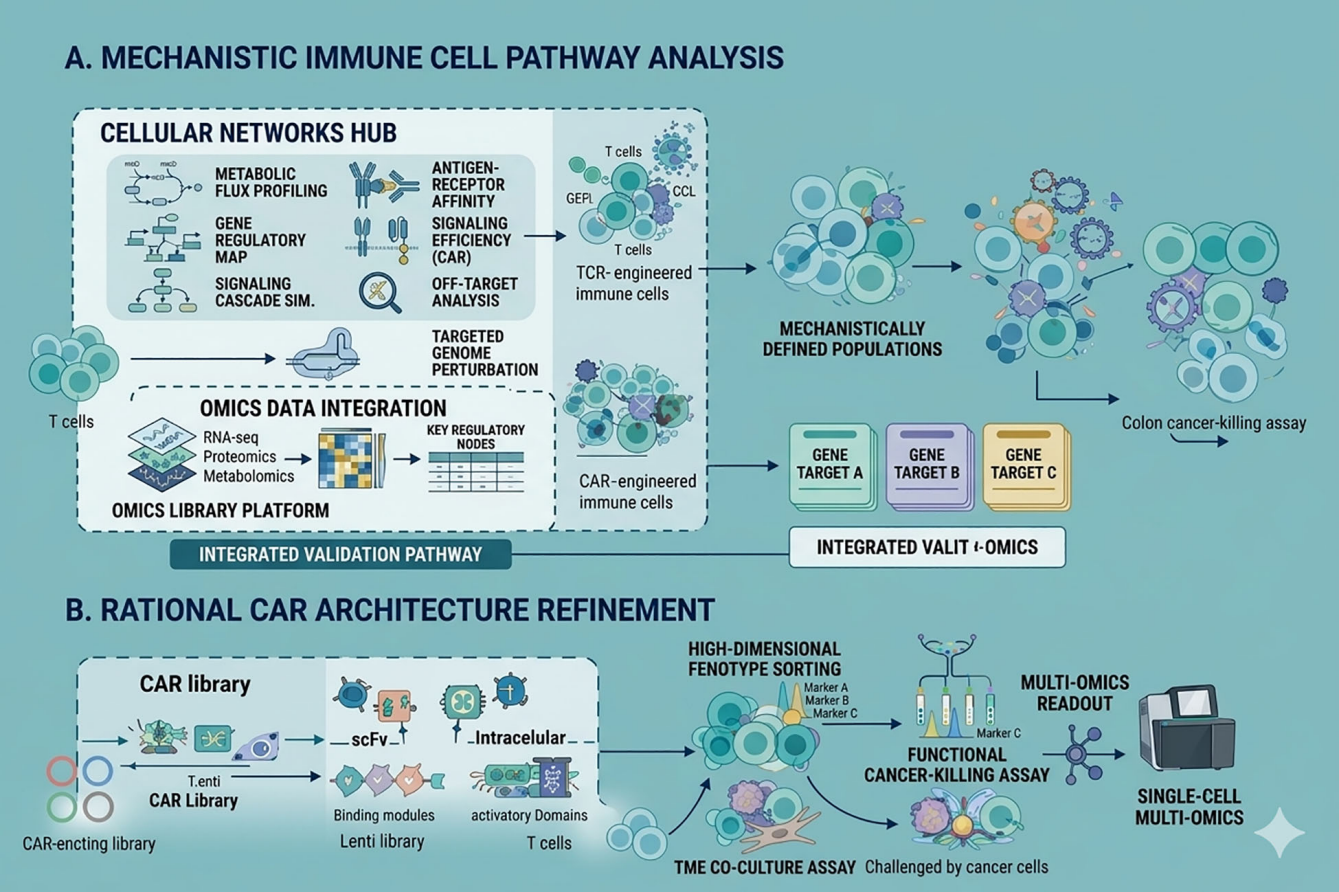
Direct screening of adoptive T cells and CAR structures. **(A)** The development of TCR-T and CAR-T libraries has shifted toward site-specific engineering at the TRAC locus to ensure more uniform expression and function. The ModPoKI system facilitates this via electroporation-mediated knock-in of functional modules, whereas the CLASH platform utilizes AAV-delivered CRISPR libraries to achieve simultaneous CAR integration and gene knockout. These integrated workflows simplify the identification of potent therapeutic candidates during tumor-killing assays. **(B)** To optimize the intracellular signaling architecture of CARs, researchers employ expansive ICD libraries containing diverse stimulatory and inhibitory motifs. After lentiviral-mediated expansion in T cells, these variants undergo rigorous functional screening for proliferation and effector function. The most resilient and cytotoxic configurations are then decoded via sequencing, providing a blueprint for next-generation CAR designs.

Refining the interaction between engineered T cells and the tumor antigenic landscape is essential for successful adoptive therapy. Given the risk of off-tumor (OTOT) effects when targeting TAAs, researchers are increasingly focusing on the modular design of CARs to achieve optimal affinity (57). By utilizing ‘light chain shuffling’ to diversify targeting scFvs, it is possible to identify receptors that distinguish between high-density tumor antigens and low-density healthy tissue expression. For example, reducing CD38-CAR affinity by three orders of magnitude has been shown to improve specificity in treating multiple myeloma (58). Integrating negative and positive screening through Combinatorial Cellular Libraries (CCL) further streamlines the identification of high-performance CAR designs (59). Moreover, modifying the complementary determining region of HER2^+^ CAR-T’s variable heavy chain, while monitoring CAR expression, antigen recognition, and signal transduction, demonstrates the importance of CAR structural design in affecting affinity and cytotoxicity, thereby reducing off-tumor (OTOT) effects (60) (**Figure 3A**).

Intracellular domains (ICDs) play a crucial role in regulating CAR-T cell behavior and typically include fundamental components such as the immunoreceptor tyrosine-based activation motif (ITAM) from CD3ζ, co-stimulatory domains like CD28 and 4-1BB, as well as other activating or inhibitory signals. The combination of these signaling domains can significantly impact the therapeutic efficacy of T cells. However, the process of designing and testing each CAR-T construct individually can be quite labor-intensive (61). The development of standardized, high-throughput libraries has revolutionized the identification of functional CAR motifs. These orthogonally designed libraries allow for the simultaneous testing of multiple stimulatory and inhibitory signals within a single cell pool. Through the assessment of persistence and tumor-killing efficiency, coupled with downstream sequencing, optimal therapeutic candidates can be isolated (62–65). Notable advancements include the CARPOOL technique for CD19-targeted therapy and the speedingCARs platform for HER2+ malignancies, the latter of which provides deep mechanistic insights via single-cell sequencing technologies (64). These integrative screening methodologies represent a paradigm shift in cellular engineering, offering a robust template for the optimization of diverse adoptive cell therapies, including those utilizing TCR-T or innate immune cells like macrophages and NK cells (62) (**Figure 3B**) (**Table 2**).

**Table 2:** Representative studies of genetic screening for TCR-T and CAR-T cells.

Screening method	Screening cells	Screening model	Construction of TCR-T or CAR-T	Library design	Screening strategies	Readout	Screened target	Function of Screened target	Ref.
CRISPR KO	NY-ESO-1 TCR-T cells	*in vitro*	Lentivirus transduction	Cytotoxic T cell gene pool	TCR stimulation, FACS sorting CD107a expression	Comparison of CD107a^+^ and CD107a^-^	SNX9	Deletion of SNX9 decreases PLCγ1, Ca2+, and NFATc2-mediated T cell signaling and reduces expression of NR4A1/3 and TOX	(52)
CRISPR KO	HER2 CAR-T cells	*in vitro*	Lentivirus transduction	Genome-wide libraries	Glioblastoma stem cells and CAR-T Co-culture, FACS sorts PD1 expression	Comparison of PD1^+^ and PD1^-^ groups	TLE4 IKZF2	Induce exhaustion responses through transcriptional process	(53)
CRISPR KO	GD2 CAR-T cells	*in vitro*	Retrovirus transduction	Genome-wide libraries	Co-cultured with GD2^+^ tumor cells, FACS sorted TNF and IL-2 expression	Compare TNF^+^/^-^ with IL-2^+^/^-^ groups	MED12	MED12 deletion enhanced antitumor activity and sustained the effector phenotype in CAR- T cells	(54)
CRISPR KO	CD22 CAR-T cells	*in vitro* *in vivo*	CLASH platform	Descartes library	Co-cultured with the NALM6 cell line NALM6-GL mice were injected into the body, and the changes of T cells over time were observed	T cells were compared over three time periods	PRDM1	The PRDM1 Δexon3 mutant improves the persistence of CAR-T cells	(55)
CRISPR KI	NY-ESO-1 TCR-T cells, CD19 CAR-T cells	*in vitro*	ModPoKI platform	TF library and surface receptor library	TCR was repeatedly stimulated to observe T cell changes	Compare T cells over different time periods	BATF TFAP4	Overexpressed BATF and TFAP4 co-occupy and regulate key gene targets to reprogram T cell function	(56)

SNX9, sorting nexin-9; TLE4, Transducin Like Enhancer of Split 4; IKZF2, Ikaros Family Zinc Finger Protein 2;MED12, Mediator Complex Subunit 12 PRDM1, PR/SET Domain 1; BATF, Basic Leucine Zipper ATF-Like Transcription Factor; TFAP4, Transcription Factor AP-4.

The field is currently undergoing a fundamental transition from bulk-population CRISPR screens to multi-modal precision perturbations. Traditional screens, while robust in identifying ‘hits,’ often obscure the stochastic nature of T-cell responses. Emerging trends now favor the integration of single-cell RNA sequencing with CRISPR (e.g., CROP-seq or Perturb-seq), allowing researchers to map the transcriptional trajectories of engineered T cells with unprecedented resolution. For instance, perturbing regulators like TET2 or REGNA1 no longer yields a binary outcome but reveals a nuanced shift in differentiation states toward stem-like memory phenotypes. Furthermore, a significant trend is the adoption of base editing and prime editing to circumvent the chromosomal instability and unintended translocations associated with Cas9-induced double-strand breaks (DSBs). This shift toward ‘scarless’ engineering directly addresses the stringent safety requirements for clinical-grade cellular products.

## 4 Genetic screening conducted on tumor cells in response to T cell pressure

Integrating CRISPR knockout screens across various tumor histologies reveals a shared “Interactome” of resistance. Our synthesis identifies IFN-γ signaling and the Antigen Presentation Machinery (APM) as universal hubs of immune evasion. The high frequency of these hits across disparate screening platforms indicates a fundamental “blind spot” in monotherapeutic CAR-T approaches. Consequently, these findings necessitate a shift in ACT design—moving away from single-target optimization toward “Logic-Gated” or multi-antigen targeting strategies specifically designed to circumvent the adaptive resistance circuits identified by HTS.

### 4.1 Antigen presentation with a targeted approach

To address the challenges of primary and acquired resistance in immunotherapy, high-throughput functional genomics provides a comprehensive platform for target discovery (66). By simulating the T-cell-targeted environment, researchers can identify tumor-intrinsic factors that contribute to resistance under immune challenge. This process involves the introduction of gene-editing libraries into tumor cells, followed by co-culture with effector T cells to identify ‘escapee’ genotypes. The use of orthotopic or systemic *in vivo* screens in various mouse strains further elucidates the regulatory landscape of the tumor microenvironment. Ultimately, such screening strategies provide a translational bridge between mechanistic discovery and therapeutic development, enabling the identification and validation of actionable targets and facilitating the development of both small-molecule inhibitors and biologic agents that potentiate T-cell-based immunotherapies.

In adoptive cell therapies, especially those involving engineered TCR-T and CAR-T, the precise interaction between tumor cell surface antigens and the engineered TCRs or antibodies on T cells is crucial. Strategies to improve antigen presentation and prevent antigen downregulation are important for overcoming resistance. Following stimulation with post-MHC (Major Histocompatibility Complex) antibodies or through co-culturing, direct screening is conducted based on the expression levels of MHC molecules and other tumor-associated antigens (TAAs). Subsequent sequencing after FACS sorting helps identify regulatory factors associated with both positive and negative antigen expression (67–74).

The landscape of tumor antigenicity is dynamically regulated by transcriptional and epigenetic factors. Using high-throughput sgRNA libraries, researchers established that the PRC2-MHC-I APP axis is a major contributor to immune escape, while the CtBP complex serves a similar role for MHC-II (67). Adhesion-mediated co-stimulation, specifically via the CD58/CD2 interaction, is further governed by CMTM6, which balances stimulatory and inhibitory signals (75–78). In CAR-T applications, CRISPRa and knockout screens have been used to stabilize antigen levels. For example, targeting NUDT21 prevents the loss of CD19 in BCP-ALL, maintaining therapeutic sensitivity (74). Similarly, the discovery that T-cell activation hinges on BTN2A1/BTN3A1 complex recognition has paved the way for therapies that target these butyrophilins to enhance innate-like anti-cancer immunity (72, 73).

### 4.2 Intrinsic mechanisms of resistance

Beyond antigen downregulation, tumor cells exhibit various other immune escape and resistance mechanisms. Moreover, the Fas/FasL and tumor necrosis factor-related apoptosis-inducing ligand (TRAIL)/TRAILR pathway create the death-inducing signaling complex (DISC), triggering the downstream Caspase cascade to induce tumor cell apoptosis, marking another key aspect of tumor immunity (79). Continuous IFN-γ or TNF stimulation in tumor cells with introduced sgRNA enables identifying regulatory factors in surviving tumor cells that are sensitive or resistant to IFN-γ or TNF pathways (27, 80, 81). Coelho et al. employed base editing screening to pinpoint mediators in colorectal cancer either sensitive or resistant to IFN-γ, leading to the identification of numerous predictable missense mutations that modify IFN-γ pathway activity (27). Freeman and team, via combined IFN-γ and TNF stimulation screening, uncovered that the linear ubiquitin chain assembly complex(LUBAC) catalytic subunit HOIP hinders intrinsic and extrinsic apoptotic pathways, thus diminishing tumor immunity (80–82).

Researchers compare tumor and T cell co-cultures both *in vitro* and *in vivo* to analyze the impact of treatments on gene expression, as well as to contrast conditions in immunocompetent versus immunodeficient mice (83–100). They utilize Gene Set Enrichment Analysis (GSEA) or functional validation experiments to indirectly identify or confirm upstream factors involved in targeted antigen presentation and intrinsic resistance mechanisms. High-throughput screening continues to elucidate the genetic drivers of immune resistance, particularly those involving cytokine signaling and antigen processing (101–104). Using CRISPRa libraries in melanoma, investigators unmasked IL10RB-DT as an lncRNA that blunts IFN-γ-mediated signaling (83). In murine lung cancer, the antigen ADAM2 was found to restructure the TME; it inhibits Type I/II IFN and TNF-α pathways while simultaneously reducing the expression of exhaustion-related markers like LAG-3 and TIM-3 (88, 101).This screening also highlighted a stark contrast in solid tumor resistance, where IFN-γ receptor signaling promotes CAR-T resistance in glioblastoma, unlike in hematologic models. Finally, the development of DNT-based therapies is being propelled by the discovery of the SAGA deubiquitination complex as a negative regulator of cytotoxicity against AML, offering new avenues to optimize these allogeneic ‘off-the-shelf’ products (105–107).

High-throughput screening has not only identified critical immune effectors but also unveiled profound biological dichotomies. A premier example is the IFN-γ signaling paradox. While multiple genome-wide CRISPR screens—particularly those in melanoma and glioblastoma models—have categorized IFN-γ receptor components as essential sensitizers for T-cell mediated killing, a synthesized analysis of recent data suggests that chronic IFN-γ exposure can dually act as a driver of adaptive resistance. This resistance manifests not only through the canonical upregulation of PD-L1 but also via non-canonical pathways involving epigenetic remodeling that renders tumor cells recalcitrant to ferroptosis and apoptosis. Such a paradox implies that therapeutic strategies aimed at constitutively amplifying cytokine signaling may inadvertently trigger compensatory escape mechanisms. Consequently, future screening paradigms must transition from static ‘kill-or-survive’ endpoints to time-resolved kinetic assays to capture these transient regulatory shifts.

## 5 Genetic screening utilized for potential combination strategies

### 5.1 Immune checkpoint blockade

Immune checkpoint blockade (ICB) has transformed cancer immunotherapy by releasing inhibitory signals that constrain T-cell activation, most prominently through antibodies targeting PD-1 and CTLA-4. Although these therapies can restore endogenous antitumor immunity, a large fraction of patients exhibit primary or acquired resistance, highlighting the need to better understand the molecular determinants governing T-cell responsiveness within the tumor microenvironment (108).

High-throughput functional genomic screening has emerged as a powerful strategy to systematically identify genetic programs that regulate tumor susceptibility to T-cell–mediated cytotoxicity. In particular, pooled CRISPR screens conducted in tumor–T cell co-culture systems or *in vivo* tumor models have revealed tumor-intrinsic pathways that influence antigen presentation, interferon signaling, and immune evasion. These approaches have identified key modulators such as IFN-inducible non-classical MHC molecules (e.g., Qa-1b/HLA-E), which can attenuate T-cell activity and limit the efficacy of checkpoint blockade (109–113). By quantifying the enrichment or depletion of sgRNAs following immune pressure, these screens enable the identification of genes that either sensitize tumors to immune attack or facilitate immune escape (113).

Importantly, these discoveries provide a mechanistic foundation for combining ICB with adoptive T-cell therapies. Genetic screening can identify tumor-intrinsic regulators that suppress the activity of both endogenous and engineered T cells, thereby revealing therapeutic targets whose inhibition may simultaneously enhance checkpoint blockade and the cytotoxic function of transferred T cells. For example, regulators of inflammatory signaling pathways, such as PRMT1 and RIPK1, have been shown to influence tumor susceptibility to immune-mediated killing; pharmacological inhibition of these pathways significantly improves the antitumor activity of checkpoint-based regimens in preclinical models (109–118). Thus, functional screening serves not only to elucidate resistance mechanisms to checkpoint inhibitors but also to guide rational combination strategies that integrate adoptive T-cell therapies with checkpoint blockade to amplify antitumor immunity (119–121).

### 5.2 BiTE therapy

Bispecific T-cell engagers (BiTEs) represent another major strategy for harnessing cytotoxic T cells against malignant cells. By simultaneously binding CD3 on T cells and tumor-associated antigens on cancer cells, BiTE molecules physically bridge effector T cells and tumor targets, triggering T-cell activation and cytolytic synapse formation independent of conventional MHC-mediated antigen presentation (122). Despite their clinical success in hematologic malignancies, BiTE therapies often encounter resistance driven by antigen loss, impaired immune synapse formation, or tumor-intrinsic survival pathways (123).

Functional genomic screening has proven particularly valuable in dissecting the molecular basis of resistance to BiTE-mediated killing. CRISPR-based screens performed in tumor–T cell co-culture systems under BiTE pressure can systematically identify genes that regulate tumor recognition, immune synapse stability, and susceptibility to T-cell cytotoxicity. For example, screening studies have demonstrated that loss of CD58—frequently associated with PAX5 alterations—confers resistance to the anti-CD19 BiTE blinatumomab through disruption of the CD2–CD58 co-stimulatory axis. Similarly, genome-wide screens in acute myeloid leukemia models treated with CD123-directed BiTE constructs have revealed that defects in interferon-γ signaling significantly impair T-cell–mediated tumor clearance.

These findings are highly relevant to adoptive T-cell therapies, as many resistance mechanisms uncovered in BiTE contexts also influence the activity of engineered T cells such as CAR-T or TCR-T cells. Because both modalities ultimately rely on effective cytotoxic T-cell engagement with tumor targets, screening-derived insights can inform strategies to enhance the performance of therapeutic T cells (124, 125). For instance, genes identified as mediators of immune synapse disruption or antigen escape may represent targets for genetic engineering of adoptively transferred T cells, or for combination therapies that restore tumor susceptibility to T-cell attack. Consequently, functional screening provides a framework for integrating BiTE-based approaches with adoptive T-cell therapies to overcome shared resistance mechanisms (126).

### 5.3 Modulating the TME

**Figure 4:**
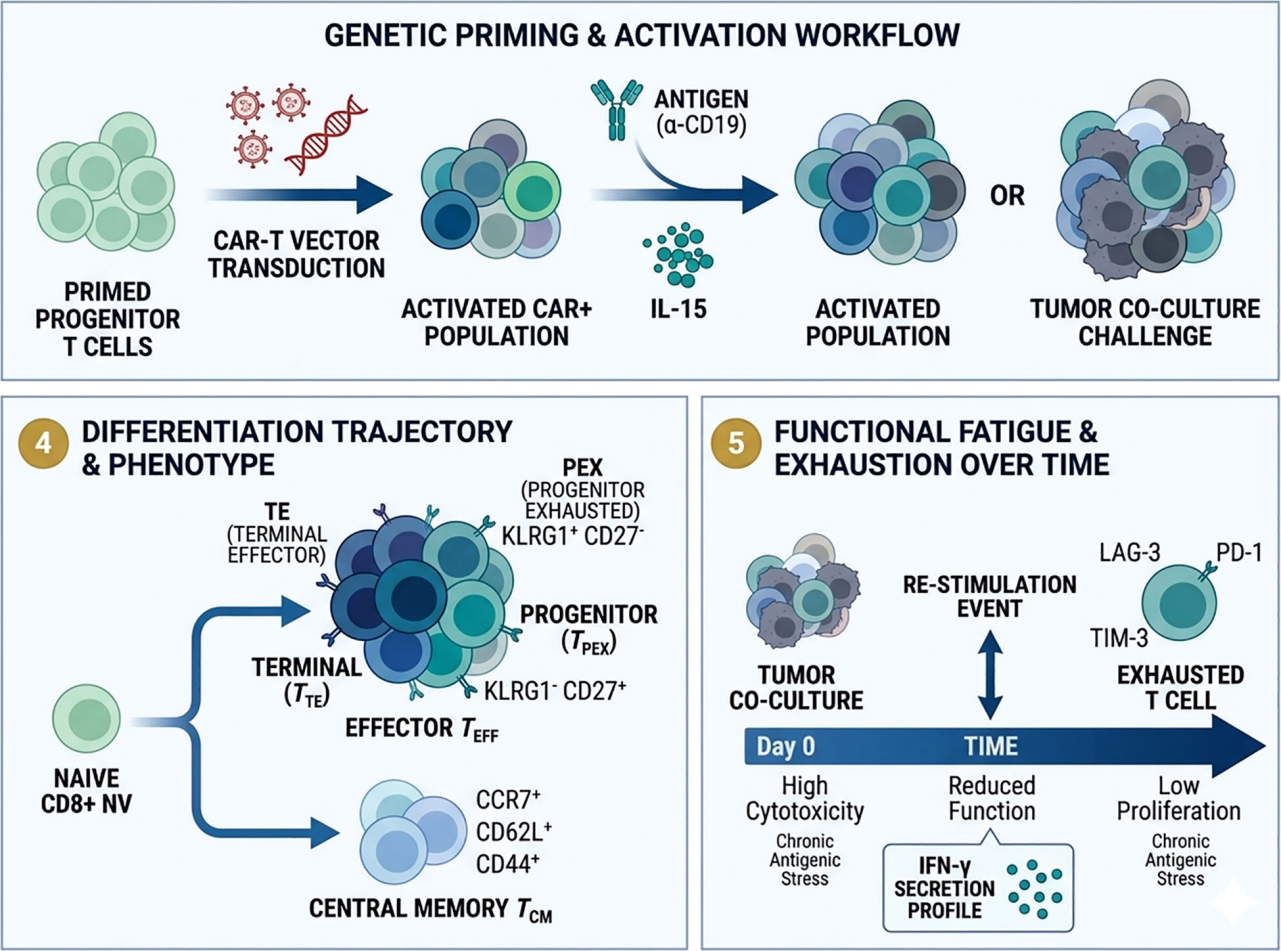
Genetic screening for modulating the TME. Macrophages play a dual role by either activating T cell functions or directly targeting tumors with cytotoxic effects. Screening macrophages involves isolating them based on their expression of MHC-II, CD40, and PD-L1 following IFN-γ stimulation to assess their ability to present antigens, as well as measuring TNF-α levels to evaluate their cytotoxic capabilities. Dendritic cells (DCs) are the key antigen-presenting cells that stimulate T cell functions. Bone marrow-derived DCs (BMDCs) are harvested from mice, and their antigen-presenting capacity is assessed through screening for CD86 and PD-L1 expression. CD4+ T cells can differentiate into various subsets, including TH1, TH2, and Tregs, influenced by different cytokines. CD4+ T follicular helper (Tfh) cells are particularly important for promoting B cell proliferation and antibody production. *In vivo* screening of naïve CD4+ T cells allows for the identification of Tfh cells via the markers CXCR5+SLAM−. Regulatory T cells (Tregs) exert a negative influence on T cell cytotoxicity, primarily through secreted factors such as TGF-β, IL-10, and IL-35. The identification of factors regulating Tregs can be achieved through screening for FOXP3 expression.

The TME plays a crucial role in tumor growth, metastasis, and prognosis, featuring immune cells with diverse functions that exert dual effects in adoptive T cell therapy. On one hand, the TME includes immune-activating cells like APCs and T follicular helper cells (Tfh), which can trigger potent immune cytotoxicity beneficial for tumor treatment. On the other hand, inhibitory cells in the TME, such as regulatory T cells (Tregs) and their secreted soluble factors, create an immunosuppressive environment that impedes T cell efficacy. High throughput genetic screening to identify targets in macrophages, dendritic cells, CD4+ T cells, and Treg cells within the TME can indirectly enhance the tumor-killing efficacy of adoptive T cell therapy (**Figure 4**).

The TME plays a central role in shaping the efficacy of adoptive T-cell therapies by regulating immune cell recruitment, activation, and persistence. Within the TME, multiple stromal and immune populations—including macrophages, dendritic cells, and regulatory T cells—can either support or suppress antitumor immune responses. Dissecting the regulatory networks governing these cell populations is therefore critical for identifying therapeutic targets capable of enhancing T-cell–mediated tumor destruction.

High-throughput genetic screening in immune and stromal cells has provided unprecedented insights into the molecular circuits controlling immune regulation within the TME (127–129). In macrophages, for example, CRISPR-based perturbation studies have identified regulators of antigen presentation and inflammatory signaling that influence the ability of macrophages to stimulate cytotoxic T-cell responses. For example, genome-wide CRISPR knockout screens performed in antigen-presenting cells stimulated with IFN-γ identified GSK3β and MED16 as key regulators of MHC class II expression. In these screens, cells were sorted according to surface MHC-II levels following interferon stimulation, and sgRNA enrichment analysis revealed genes whose disruption impaired interferon-induced antigen presentation. Mechanistically, GSK3β modulates interferon-responsive transcriptional programs, whereas MED16, a component of the mediator complex, is required for the transcriptional activation of CIITA-dependent MHC-II expression (130–138). Targeting these pathways may promote a more immunostimulatory macrophage phenotype that supports the activity of adoptively transferred T cells.

Similarly, functional screening of regulatory T cells has revealed key transcriptional and metabolic programs that sustain their immunosuppressive state (139). Screens focusing on Foxp3-dependent regulatory circuits have identified modifiers such as Slc35c1 and Usp22 that stabilize Treg suppressive function, while metabolic regulators such as MTHFD2 have been shown to control Treg differentiation and persistence (140, 141). Disrupting these pathways may selectively destabilize Tregs within the tumor microenvironment, thereby relieving suppression of cytotoxic T cells (142).

Collectively, these discoveries highlight the power of functional screening to identify actionable nodes within the TME that influence the effectiveness of adoptive T-cell therapies (143, 144). By targeting immunosuppressive pathways or reprogramming supportive immune populations, combination strategies that integrate TME-modulating interventions with adoptive T-cell therapy may significantly enhance antitumor efficacy.

### 5.4 Synthesized perspectives and methodology blind spots

A critical synthesis of HTS data identifies a significant divergence between *in vitro* and *in vivo* outcomes. Many “hits” that maximize immediate cytotoxicity in 2D co-culture fail to translate into therapeutic efficacy in 3D models or clinical settings. This discrepancy highlights a critical methodological blind spot: current screening platforms often fail to account for the physical and mechanical barriers of the extracellular matrix (ECM) and the high-pressure interstitial fluid of solid tumors. Future HTS paradigms must therefore evolve from simple viability screens to “Spatial-Functional” screens that incorporate tumor organoids and microfluidic systems to better simulate the biophysical resistance encountered in human patients.

### 5.5 Critical gaps and strategic blind spots

Despite technological maturation, several systemic blind spots persist that hinder the clinical translation of HTS-derived targets. First is the spatial-contextual void: most HTS assays are conducted in liquid co-cultures or 2D monolayers, which fail to recapitulate the physical impedance of the extracellular matrix (ECM) and the interstitial fluid pressure within solid tumors. This likely explains the high attrition rate of targets that exhibit potency *in vitro* but fail in human physiological environments. Second is the decoupling of safety and efficacy evaluations. Current screening strategies remain heavily biased toward ‘potency enhancement’ while lacking negative-selection screens designed to proactively identify targets that might trigger cytokine release syndrome (CRS) or off-tumor toxicities. Addressing these gaps requires the development of high-fidelity screening platforms that incorporate patient-derived organoids (PDOs) and 3D microfluidic ‘tumor-on-a-chip’ systems to provide a more predictive translational bridge.

### 5.6 Navigating the preclinical-clinical gap: successes, failures, and translational hurdles

Although high-throughput screening (HTS) has generated an expansive catalogue of candidate regulators capable of enhancing T-cell function, the clinical translation of these discoveries remains the ultimate benchmark of success. Moving from simplified experimental systems—such as *in vitro* co-culture platforms or murine tumor models—to the human physiological environment is frequently constrained by what can be described as a translational funnel. This process is characterized by a steep attrition rate driven by unforeseen toxicities, incomplete recapitulation of tumor biology in preclinical models, and the adaptive heterogeneity of the human tumor microenvironment (TME). Understanding the mechanisms underlying these translational barriers is therefore essential for converting screening-derived insights into clinically effective therapies.

#### 5.6.1 Successful clinical intersections and emerging trials

Some of the most promising translational advances have emerged from screening strategies that identify master regulators of T-cell fate and persistence. A notable example arises from the ModPoKI platform, which revealed the transcription factors BATF and TFAP4 as synergistic drivers of sustained T-cell function. These regulators appear to reinforce transcriptional programs associated with metabolic fitness and resistance to exhaustion. On the basis of these findings, CAR-T cell products engineered to overexpress such transcriptional regulators are currently entering early-phase clinical evaluation, with the aim of improving T-cell durability in patients with refractory solid tumors.

In parallel, HTS approaches have contributed to the structural optimization of CAR architecture itself. Screening of scFv libraries targeting CD19 has enabled the identification of antibody fragments with optimized binding kinetics and reduced tonic signaling. This affinity-tuning strategy helps limit excessive cytokine release while preserving potent antitumor activity. The principle of calibrated antigen binding has subsequently been incorporated into several clinically successful second-generation CD19 CAR-T designs, demonstrating how HTS can inform both molecular target selection and receptor engineering (**Table 3**).

**Table 3:** Clinical landscape and translational status of HTS-Derived targets.

Targeted gene/mechanism	HTS platform	Preclinical evidence	Clinical status/NCT ID	Translational challenges & analysis
BATF/TFAP4	ModPoKI (KI)	Enhanced persistence and metabolic fitness	Phase I/II (e.g., NCT05633914)	Success: Moving toward multi-target “modular” engineering.
RASA2	CRISPR KO	Significant increase in MAPK signaling and killing	Preclinical/IND Stage	Risk: High potential for hyper-activation; requires “safety-switch” integration.
PRDM1 (Δexon3)	CLASH (KO)	Improved long-term memory *in vivo*	Preclinical	Challenge: Difficulty in ensuring precise splice-variant editing in clinical-scale manufacturing.
PD-1 Disruption	Genome-wide KO	Reversal of exhaustion in mouse models	Multiple Trials (e.g., NCT03706326)	Observation: Mixed clinical results; human TME often utilizes LAG-3/TIM-3 as compensatory pathways.
HER2 Affinity	scFv Library	Optimized binding for low-antigen density	Discontinued/Revised	Failure: Fatal OTOT toxicity; highlights the need for “Safety-First” filters in HTS.

#### 5.6.2 Critical analysis of “lost in translation” cases

Despite these successes, many targets that exhibit strong efficacy in preclinical models fail to translate into clinical benefit. One of the most prominent barriers is the potency–toxicity trade-off. High-affinity CAR constructs targeting tumor-associated antigens such as HER2 or CEA, which were optimized through HTS to maximize tumor cell lysis, have historically resulted in severe or even fatal on-target, off-tumor (OTOT) toxicities in patients due to low-level antigen expression in healthy tissues.

In addition, immune checkpoint–related modifications have produced mixed outcomes in clinical settings. For example, disruption of the inhibitory receptor PD-1 has demonstrated striking therapeutic effects in murine tumor models; however, clinical trials of PD-1–deficient CAR-T cells have yielded heterogeneous efficacy. Increasing evidence suggests that this discrepancy arises from adaptive resistance networks within the human TME, where tumor and stromal cells dynamically upregulate compensatory inhibitory ligands such as LAG-3 or TIM-3. These adaptive resistance hubs, which have been highlighted in tumor-intrinsic screening studies, are often insufficiently represented in conventional two-dimensional or even three-dimensional preclinical screening systems.

#### 5.6.3 Future requirement: safety-informed design

Reducing the high attrition rate during clinical translation will require a fundamental shift in screening philosophy. Future HTS platforms should evolve beyond potency-focused discovery toward integrated assays capable of simultaneously evaluating therapeutic efficacy and safety. Such “safety-informed” screening strategies may incorporate negative-selection platforms using human-derived healthy organoids, as well as cross-reactivity assessments in non-human primate (NHP) models to detect potential toxicities prior to first-in-human studies.

By integrating these safety-oriented filters early in the discovery pipeline, HTS frameworks may better capture the complex physiological constraints that shape therapeutic performance in patients. Ultimately, addressing these translational blind spots will improve the predictive power of preclinical screening and facilitate the development of clinically durable synthetic immune therapies.

### 5.7 Perturb-seq enables high-resolution dissection of T-cell regulatory programs

A rapidly emerging platform that further expands the scope of functional genomic screening is Perturb-seq, which integrates CRISPR-mediated genetic perturbations with single-cell transcriptomic profiling. By linking defined genetic perturbations to high-dimensional transcriptional states in thousands of individual cells simultaneously, Perturb-seq enables systematic reconstruction of gene regulatory networks governing immune cell function. In T cells, this approach has begun to reveal the molecular circuits that control activation, differentiation, cytokine production, and exhaustion. Importantly, single-cell perturbation screens can resolve heterogeneous T-cell states and lineage trajectories that are not captured by bulk screening strategies, thereby providing deeper insight into the determinants of T-cell persistence and functional fitness. When applied to tumor–immune co-culture systems or *in vivo* models, Perturb-seq also enables the identification of tumor-intrinsic pathways that shape susceptibility to T-cell–mediated cytotoxicity. These capabilities position Perturb-seq as a powerful framework for discovering genetic modifications and regulatory programs that can be harnessed to engineer next-generation adoptive T-cell therapies with improved durability and antitumor efficacy.

## 6 Applications of non-genetic screening in adoptive T cell therapies

### 6.1 Screening of small-molecule compounds

The integration of small-molecule libraries with adoptive T-cell screening has redefined the search for combinatorial immunotherapies. This high-throughput methodology allows for the evaluation of drug-induced changes in T-cell cytotoxicity and tumor vulnerability (145). Research utilizing a diverse library of 526 molecules in CD19 CAR-T systems pinpointed SMAC mimetics as critical enhancers of anti-tumor efficacy. Subsequent genetic screening revealed that these mimetics act by sensitizing tumor cells to apoptosis via upregulated death receptor pathways. In solid tumor models, erlotinib was found to synergize with CD8+ T cells to overcome resistance in ovarian cancer, suggesting that targeted inhibitors can act as immunomodulators to enhance adoptive cell therapy outcomes (146).

Immune cells and cancer cells share many critical metabolic pathways, leading to intense competition for nutrients within the tumor microenvironment (TME). Metabolic reprogramming in both tumor cells and T cells is a key aspect of tumor immunity, particularly in pancreatic ductal adenocarcinoma (PDAC). Therefore, genetic screening is employed to investigate fundamental metabolic pathways involved in pancreatic tumor progression and TME-induced dependencies, aiming to identify potential therapeutic targets (147, 148).

### 6.2 Targeted delivery system screening

**Figure 5:**
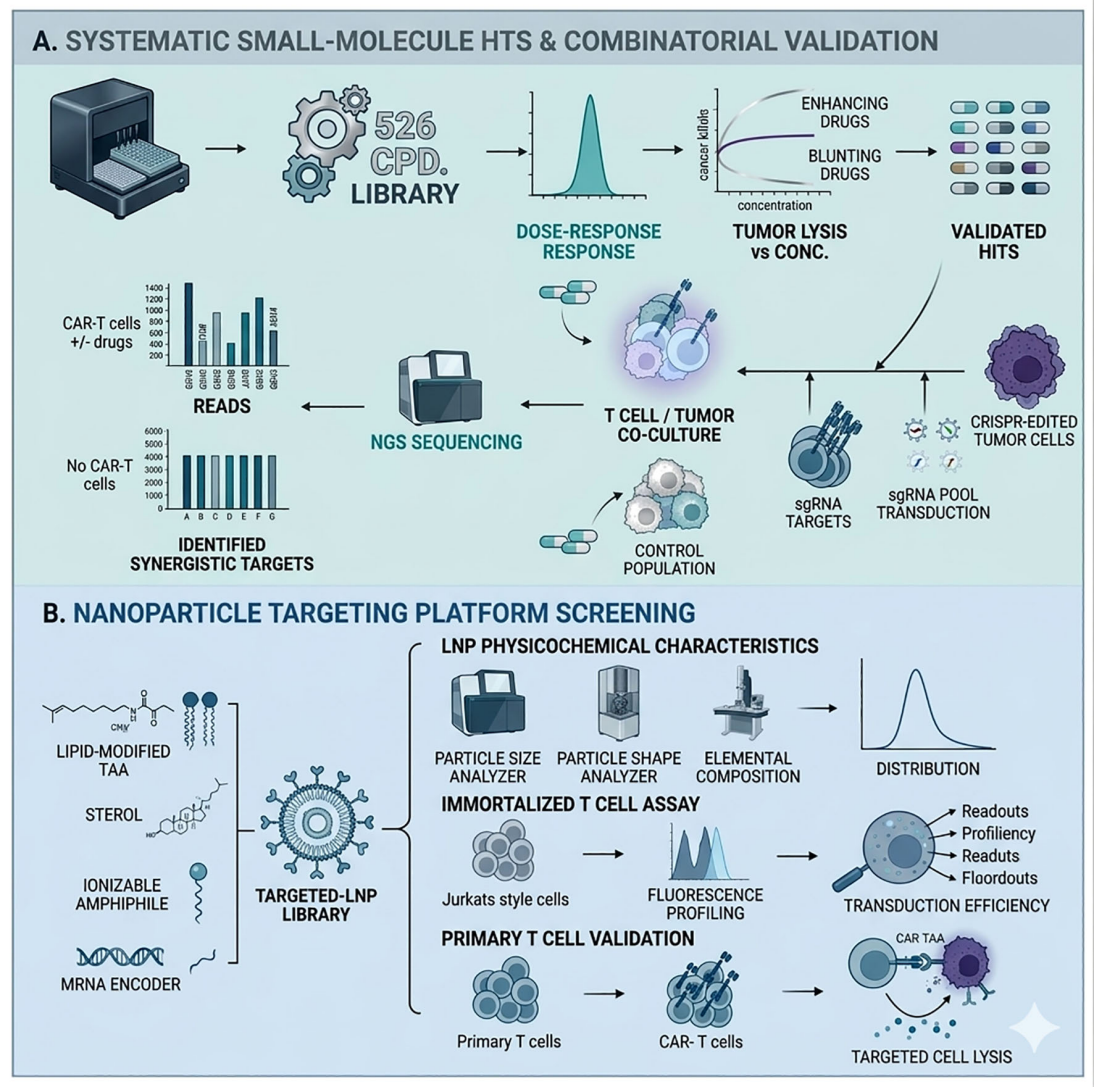
Applications of non-genetic screening in adoptive T Cell therapies. **(A)** The pipeline for identifying synergistic immunotherapies begins with the screening of small-molecule libraries against CAR-T and tumor cell co-cultures. Tumoricidal assays pinpoint specific drugs that enhance CAR-T activity, which are then further analyzed using tumor cells transfected with sgRNA libraries. This dual-screening method is essential for clarifying the molecular mechanisms that drive drug-immunotherapy synergy. **(B)** mRNA-mediated CAR-T engineering relies on the optimization of LNP molecular architecture. By systematically varying the ratios of auxiliary lipids, cholesterol, and ionizable lipids through an orthogonal library, researchers identified formulations with superior T-cell targeting. These LNPs were characterized for their physical properties and tested in Jurkat models before being applied to primary T cells to demonstrate clinical-grade tumor-killing capabilities.

The transition from viral-mediated genomic integration to transient mRNA expression represents a major evolution in adoptive T-cell therapy. While viral vectors provide stable expression, they are limited by safety risks and complex manufacturing protocols (149, 150). In contrast, mRNA delivery systems—particularly those utilizing lipid nanoparticles (LNPs)—allow for the rapid, ex vivo engineering of autologous T cells without the threat of insertional mutagenesis (151). This method supports a more controlled pharmacokinetic profile and has been shown to elicit potent immune responses against various malignancies in pre-clinical models (**Figure 5A**). By improving both the safety and cost-effectiveness of T-cell engineering, mRNA technologies are poised to expand the reach of cellular immunotherapies (152, 153).

Engineering T cells with mRNA requires delivery systems that bypass hepatic accumulation and specifically engage lymphocytes. High-throughput screening of synthetic LNP formulations has emerged as a robust strategy to identify lipids with optimized T-cell organotropism (154). This process involves characterizing the efficiency of mRNA translation and the resulting lytic capacity of the engineered cells. Notably, the discovery of the C14–4 ionizable lipid provides a high-performance alternative to viral vectors for CAR-T production. By delivering CD19 CAR mRNA via C14–4 LNPs, researchers have demonstrated a scalable, non-viral method for treating B-cell malignancies while maintaining high therapeutic efficacy (155) (**Figure 5B**). For mRNA-based CAR-T engineering, lipid nanoparticles (LNPs) represent a promising non-viral delivery platform that enables transient expression of therapeutic receptors. During LNP optimization, orthogonal library design is often used to systematically vary key formulation components—including ionizable lipids, helper lipids, and cholesterol—to identify combinations that maximize delivery efficiency. Candidate LNP formulations are first evaluated through physicochemical characterization, including particle size distribution and polydispersity measured by Dynamic light scattering, surface charge (zeta potential), and structural morphology examined by Transmission electron microscopy. Encapsulation efficiency and stability of mRNA cargos are also quantified to ensure effective delivery. Subsequently, functional screening is performed in Jurkat T cells to evaluate transfection efficiency and CAR expression. Promising LNP formulations are then validated in primary human T cells, where the resulting CAR-T cells are assessed for antigen-specific cytotoxicity against tumor targets.

Moving forward, the field must transcend the “cataloging phase” of HTS. We hypothesize that the ultimate optimization of ACT lies in the Metabolic-Epigenetic Coupling Hypothesis: the notion that metabolic screening hits (e.g., PRODH2) do not merely provide fuel but act as upstream regulators of epigenetic modifications. We propose that future research focuses on “Closed-Loop Engineering,” where AI-driven HTS platforms dynamically adjust CAR-T intracellular domains based on real-time feedback from the tumor’s metabolic signature. Such a transition from discovery to an automated design-build-test cycle will be the definitive hallmark of the next decade in cellular immunotherapy.

## 7 Conclusion and future perspective: toward a roadmap for third-generation ACT

High-throughput screening (HTS) technologies have generated an unprecedented catalogue of candidate regulators capable of reshaping T-cell function. However, the field is now approaching an inflection point. Rather than continuing to accumulate isolated genetic “hits”, the next phase of adoptive T-cell therapy (ACT) will require a transition from largely stochastic discovery toward principle-guided engineering of T-cell states and circuits. Based on the emerging evidence summarized in this Review, we propose that the coming decade of ACT development will likely be shaped by three conceptual shifts, each of which can be formulated as a testable hypothesis.

### 7.1 The “synergistic modularity” hypothesis

One explanation for the limited clinical translation of many single-gene perturbations is the multi-layered resistance imposed by the solid tumour microenvironment (TME). Genetic modifications that enhance one functional dimension of T-cell activity may therefore be insufficient to overcome the combined pressures of metabolic deprivation, inhibitory signaling and epigenetic exhaustion.

We hypothesize that next-generation ACT will increasingly rely on rationally integrated functional modules, rather than isolated genetic edits. Such modular designs could combine: (i) an epigenetic stabilizing module that maintains stem-like or exhaustion-resistant transcriptional states (for example through regulators such as BATF or c-Jun); (ii) a metabolic resilience module that sustains T-cell fitness in nutrient-restricted TMEs, potentially through regulators of mitochondrial or amino-acid metabolism; and (iii) a synthetic sensing module that allows T cells to bypass tumour-intrinsic immune resistance pathways, including impaired interferon signaling. Within this framework, HTS platforms will serve not only to identify individual targets, but also to systematically uncover synergistic gene circuits that maintain robust functionality across heterogeneous tumour contexts.

### 7.2 The “transcriptional trajectory” hypothesis

Traditional screening pipelines frequently evaluate perturbations using binary phenotypic outputs, such as survival or cytotoxicity. However, clinical efficacy of ACT appears to depend less on immediate effector potency than on the ability of transferred T cells to sustain durable functional states.

We therefore propose that the most clinically relevant targets will be those that bias T cells toward a persistent-effector differentiation trajectory. Emerging single-cell perturbation technologies, including multimodal approaches such as Perturb-seq, now enable the mapping of transcriptional and epigenetic landscapes following genetic perturbations at high resolution. These approaches may reveal regulators that stabilize T-cell states characterized by sustained mitochondrial fitness, controlled proliferative potential and reduced expression of inhibitory receptors such as PD-1 or LAG-3 during chronic antigen exposure. This perspective reframes screening strategies from identifying perturbations that maximize acute cytotoxic potency toward those that shape long-term cellular fate.

### 7.3 The “*In situ* reprogramming” hypothesis

A major barrier to the broad clinical implementation of ACT remains the complexity of ex vivo cell manufacturing. The convergence of HTS with emerging non-viral delivery platforms, particularly lipid nanoparticle (LNP) systems, raises the possibility of directly reprogramming immune cells *in vivo*.

We hypothesize that systematic screening approaches could identify LNP formulations with defined tissue tropism—such as preferential delivery to secondary lymphoid organs—enabling the transient *in situ* programming of endogenous T cells. If achievable, this strategy could transform ACT from a highly individualized cellular procedure into a more scalable pharmacological intervention. Achieving this goal will require screening platforms capable of evaluating delivery efficiency, specificity and safety within physiologically relevant systems.

Taken together, these emerging directions suggest that the future of ACT will be shaped by a shift from target discovery to design principles. Addressing current blind spots—including the spatial complexity of the TME and the challenge of decoupling therapeutic efficacy from toxicity—will be critical for translating HTS discoveries into clinically robust cell therapies. Ultimately, the integration of systems-level screening, synthetic biology and advanced delivery technologies may enable the rational engineering of “synthetic” T-cell states that are metabolically resilient, transcriptionally stable and therapeutically accessible. Such advances could define the next generation of cellular immunotherapies and expand the clinical reach of ACT across diverse malignancies.
